# Thermotropic Liquid Crystals for Temperature Mapping

**DOI:** 10.3389/fbioe.2022.806362

**Published:** 2022-05-12

**Authors:** Vanja Miskovic, Elena Malafronte, Christophe Minetti, Hatim Machrafi, Carolina Varon, Carlo Saverio Iorio

**Affiliations:** ^1^ Service Chimie-Physique, Université Libre de Bruxelles, Brussels, Belgium; ^2^ GIGA-In Silico Medicine, Université de Liége, Liège, Belgium

**Keywords:** temperature sensing, infrared thermography, colorimetric sensor, temperature distribution, thermotropic liquid crystals

## Abstract

Wound management in Space is an important factor to be considered in future Human Space Exploration. It demands the development of reliable wound monitoring systems that will facilitate the assessment and proper care of wounds in isolated environments, such as Space. One possible system could be developed using liquid crystal films, which have been a promising solution for real-time *in-situ* temperature monitoring in healthcare, but they are not yet implemented in clinical practice. To progress in the latter, the goal of this study is twofold. First, it provides a full characterization of a sensing element composed of thermotropic liquid crystals arrays embedded between two elastomer layers, and second, it discusses how such a system compares against non-local infrared measurements. The sensing element evaluated here has an operating temperature range of 34–38°C, and a quick response time of approximately 0.25 s. The temperature distribution of surfaces obtained using this system was compared to the one obtained using the infrared thermography, a technique commonly used to measure temperature distributions at the wound site. This comparison was done on a mimicked wound, and results indicate that the proposed sensing element can reproduce the temperature distributions, similar to the ones obtained using infrared imaging. Although there is a long way to go before implementing the liquid crystal sensing element into clinical practice, the results of this work demonstrate that such sensors can be suitable for future wound monitoring systems.

## 1 Introduction

Although injuries, traumatic events, and surgical emergencies have been unlikely during the current space missions, their occurrence must be considered for future human space explorations. During long-lasting Space-missions, wound treatment and monitoring could become a fundamental problem, demanding more research in this area (Barratt and Pool, 2008; Cialdai et al., 2020). For example, first-degree burns can occur as a result of ultraviolet (UV) light exposure through unfiltered spacecraft windows (Barratt and Pool, 2008). Space represents a very special remote environment, and solutions developed for Space applications could inspire the creation of a better healthcare system in remote areas on-ground.

When a wound occurs, the first step is to assess its severity, which will form the basis for the following treatment. Clinical assessment of the wound is still the most common and cost-efficient method to assess wound severity. This method relies on a subjective evaluation of the wounds’ external features, such as size of the wound, wound edges, site of wound, wound bed (colour, amount of granulation tissue), presence of necrotic tissue, wound’s depth, level of exudate, and pain caused by the wound (Grey et al., 2006). The advantages of this method are that it is a rapid method and does not require specialized equipment. However, it is subjective. For instance, a nurse and clinician may have different evaluation and assessment criteria depending on their prior experiences (Nagle et al., 2020). Seeing that wound severity is usually diagnosed by a specialist, a problem appears when wound injury happens in places that lack wound care specialists, a situation common in remote areas. One way to face these problems is to develop diagnostic tools and utilities that are cost-efficient, easy-to-use, and that could support medical workers in wound diagnosis and evaluation of the healing by monitoring one or more biomarkers.

Among many clinical biomarkers, special attention has been given to pH (Shukla et al., 2007; Schreml et al., 2011; Jones et al., 2015; Power et al., 2017), oxygen (Schreml et al., 2010), and exudate composition, with the focus on matrix metalloproteinase analytes (MMPs) (Muller et al., 2008; Liu et al., 2009; Power et al., 2017). Also, temperature (Dini et al., 2015; Chanmugam et al., 2017; Jaspers et al., 2017; Power et al., 2017; Cwajda-Białasik et al., 2020) and moisture (Bishop et al., 2003; Milne et al., 2016) are the physical parameters that are frequently associated with the wound healing process. In particular, the temperature is considered as an informative parameter for all types of wounds, and it can provide information about infections of wounds, even before clinical signs appear (Power et al., 2017).

A common way to measure wound temperature is by means of Infrared (IR) thermography (Ring and Ammer, 2012). Thanks to the development of portable high-resolution affordable thermal cameras, interest in their use for wound assessment has increased. This is mainly due to their non-invasive and easily interpretable results obtained in a very short period. Various studies (Dini et al., 2015; Chanmugam et al., 2017; Jaspers et al., 2017; Martínez-Jiménez et al., 2018; Cwajda-Białasik et al., 2020; Ganon et al., 2020) have used the temperature difference between wound and healthy skin (ΔT), measured by using IR thermography, to characterize the wound status. An increase of ΔT can be related to hyperaemia, inflammation, or infection in venous leg ulcers (Dini et al., 2015; Cwajda-Białasik et al., 2020) or pressure ulcers and surgical wounds (Chanmugam et al., 2017). In burns, the temperature is often correlated to the burn depth and the burn healing time (Jaspers et al., 2017; Martínez-Jiménez et al., 2018; Ganon et al., 2020). Instead of just measuring the temperature locally, these studies demonstrated the importance of measuring the temperature distribution on the wound site and the surrounding healthy skin. Despite these promising results, IR thermography is not yet applied in daily clinical practice for wound assessment, mainly because standard guidelines and protocols have not been established yet (Shterenshis, 2017).

The field of flexible and wearable bioelectronics, capable of monitoring physiological information and assisting in proper treatment is growing exponentially (Rogers et al., 2018; Chen et al., 2020). Researchers and engineers are working on the development of new technologies for smart point-of-care systems (Zhang et al., 2016; Coppola et al., 2021). Therefore, the development of a point-of-care device for wound monitoring is more feasible. Such a device could reduce hospitalization times, the suffering of the patients, and costs (Mehmood et al., 2014; Derakhshandeh et al., 2018). Moreover, it could provide the ability to face emergency surgery, acute trauma, burns, and wounds, in remote and closed environments, such as Space. At present, wearable technology in wound care is limited to laboratory testing, and commercial wearable point-of-care systems are not broadly available. The reason could be found in the complexity of the wound healing process, the broad variety of wounds’ types, and the limited understanding of relevant wound biomarkers.

Ideally, a smart sensor for wound monitoring should have specific properties, such as 1) wearability/ability to adapt to the body shape, 2) biocompatibility, 3) high sensitivity, 4) easy-to-use, and 5) no external power. Due to these requirements, sensors based on the colourimetric approach appear as an ideal solution (Ajay et al., 2017; Isapour and Lattuada, 2018). In that respect, liquid crystals (LCs) have emerged as a promising technology for wound management sensing. The most interesting property of LCs is their structural colouration that can be manipulated by changing the external parameters, such as temperature. This makes them ideal candidates for the development of easy-to-use, label-free, and passive sensors, where the output signal is a change in colour detectable by the naked eye. LCs have a long history of being used as responsive materials in different technologies, thanks to their unique properties. Numerous studies have demonstrated the possibility to produce rapid diagnostic optical sensors for temperature (Gao et al., 2014), pH (long Chen et al., 2018), humidity (Saha et al., 2012; Zhao et al., 2019), gas (Esteves et al., 2020) and molecules (ang Niu et al., 2017; Zhang et al., 2018) based on LCs. The most widespread sensors are those for temperature, owing to the diversity and availability of thermotropic LCs.

LCs are a unique state of matter between crystalline solid and isotropic liquid. In thermotropic LCs, phase transitions from crystalline solid to smectic, cholesteric and, finally, isotropic liquid, are caused by temperature changes, and they are mainly composed of rod-like molecules. In cholesteric LCs, also known as chiral LCs, molecules are inherently chiral, and the average molecular orientation is twisted with a certain periodicity, leading to a helical structure. This structure is characterised by a helical pitch that refers to the distance over which the LC molecules undergo a whole 360° twist. The size of this pitch determines the wavelength of the reflected light. The pitch of a cholesteric LC can be of the order of magnitude that corresponds to the wavelength spectra of visible light, allowing structural colouration to occur. An increase in the temperature results in a decrease in the pitch, which causes a shift in the wavelength of the reflected light. This presents the basis of the sensing principle of cholesteric LCs (Mitov, 2012). Thermotropic LCs, intended to be used as colourimetric temperature sensors, are characterized by three parameters: the lower clearing point temperature, optical activation range, and the higher clearing point temperature (Abdullah et al., 2010). The lower clearing point temperature is the temperature at which LCs first reflect colour in the visible spectrum (red). The optical activation range is the temperature range at which thermotropic LCs actively reflect visible light. When the thermotropic LCs pass through their optical activation range, they reflect visible light from longer wavelengths (red) to shorter wavelengths (blue) as temperature increases until their higher clearing point temperatures are reached. The higher clearing point (further in the text referred as clearing point) temperature is the temperature at which thermotropic LCs stop to reflect colours in the visible spectrum. Beyond the clearing point temperature, thermotropic LCs are transparent again (Abdullah et al., 2010).

A study from 2017 (LeSar et al., 2017) investigated the possibility to use commercial thermotropic LC–coated fabric for the early detection of high-risk foot complications. Using direct visual analysis, they demonstrated that the fabric could accurately map temperatures on the surface of a hand, which supported the hypothesis that this approach can be used to develop a temperature-sensitive system to monitor complications high-risk foot. They used two different types of LC fabrics to expand the active range. The most recent prospective study (Hodorowicz-Zaniewska et al., 2020) used the Brast Tester–LC foil (Braster SA, Ozarów Mazowiecki, Poland) for the early detection of breast cancer. Despite the good performance, this contact LC thermography did not find its way into commercial use. Reasons for this include that the protocol still requires a medical specialist for its implementation, it lacks standardization, and it has been commonly replaced by contactless infrared thermography. Gao et al. (2014) were the first ones to propose a skin-like system that consists of thermotropic LCs patterned into large-scale, pixelated arrays on thin elastomeric substrates, demonstrating that such a system could be used as an epidermal temperature sensor.

This work compares the sensing ability of LCs with respect to IR thermography. To this aim, temperature mappings of surfaces of different topographies obtained using LCs sensing elements are compared against the ones obtained using IR thermography. The IR thermography is chosen as a reference since it is the most widespread technique used in wound temperature studies (Dini et al., 2015; Chanmugam et al., 2017; Jaspers et al., 2017; Martínez-Jiménez et al., 2018; Cwajda-Białasik et al., 2020; Ganon et al., 2020). This comparison will bring new insights into the possibility of using LCs-based systems for wound temperature monitoring. Some challenges in their application in clinical practice will be highlighted. Although, the final aim of the research here presented is temperature wound analysis, this paper is focused on preliminary construction and analysis of the measuring potential of the envisaged device.

## 2 Materials and Methods

### 2.1 Cholesteric Liquid Crystals

In this work, thermotropic LCs were prepared using Cholesteryl oleyl carbonate, Cholesteryl pelargonate and Cholesteryl benzoate (Sigma-Aldrich). Four thermotropic LCs were prepared, with different pitch values, by varying the concentration of the aforementioned components, as shown in Table 1. In order to mix the components appropriately, the powder mixture was heated until 60°C, at which a uniform isotropic liquid is obtained.

**TABLE 1:** Liquid crystals samples series.

Sample name	Cholesteryl oleyl carbonate (wt%)	Cholesteryl pelargonate (wt%)	Cholesteryl benzoate (wt%)
LC1	35	55	10
LC2	32.5	57.5	10
LC3	30	60	10
LC4	25	65	10

### 2.2 Spectrophotometry of LCs

The first step in the research presented here was the choice of LCs formulation that has an optical activation range in a temperature range that is useful for wound monitoring. For this purpose, transmission spectra of 4 LCs formulations (see Table 1) were measured. Spectrophotometric analysis of LCs thin films was carried out to determine the temperature range of the cholesteric liquid crystals in which they exhibit reflection peaks in the visible spectrum (390–700 nm). For this analysis, UV-Vis Spectrometer (UV3600, Shimadzu, Japan) was used. The temperature inside the spectrophotometer was controlled using a water temperature controlling system. A thin film of LCs was uniformly coated on the cell wall. A thermocouple was placed directly inside the cell on the level where the beam is passing, and the temperature was measured in real-time on the screen. During the test, the temperature was increased with steps of 0.5°C. Transmission measurements were carried out once the temperature inside the cell was stabilized. Transmittance spectra of different LCs were collected while changing the temperature from 29.9 to 44.6°C, in steps of 0.5°C.

### 2.3 Patch Design and Fabrication

**FIGURE 1:**
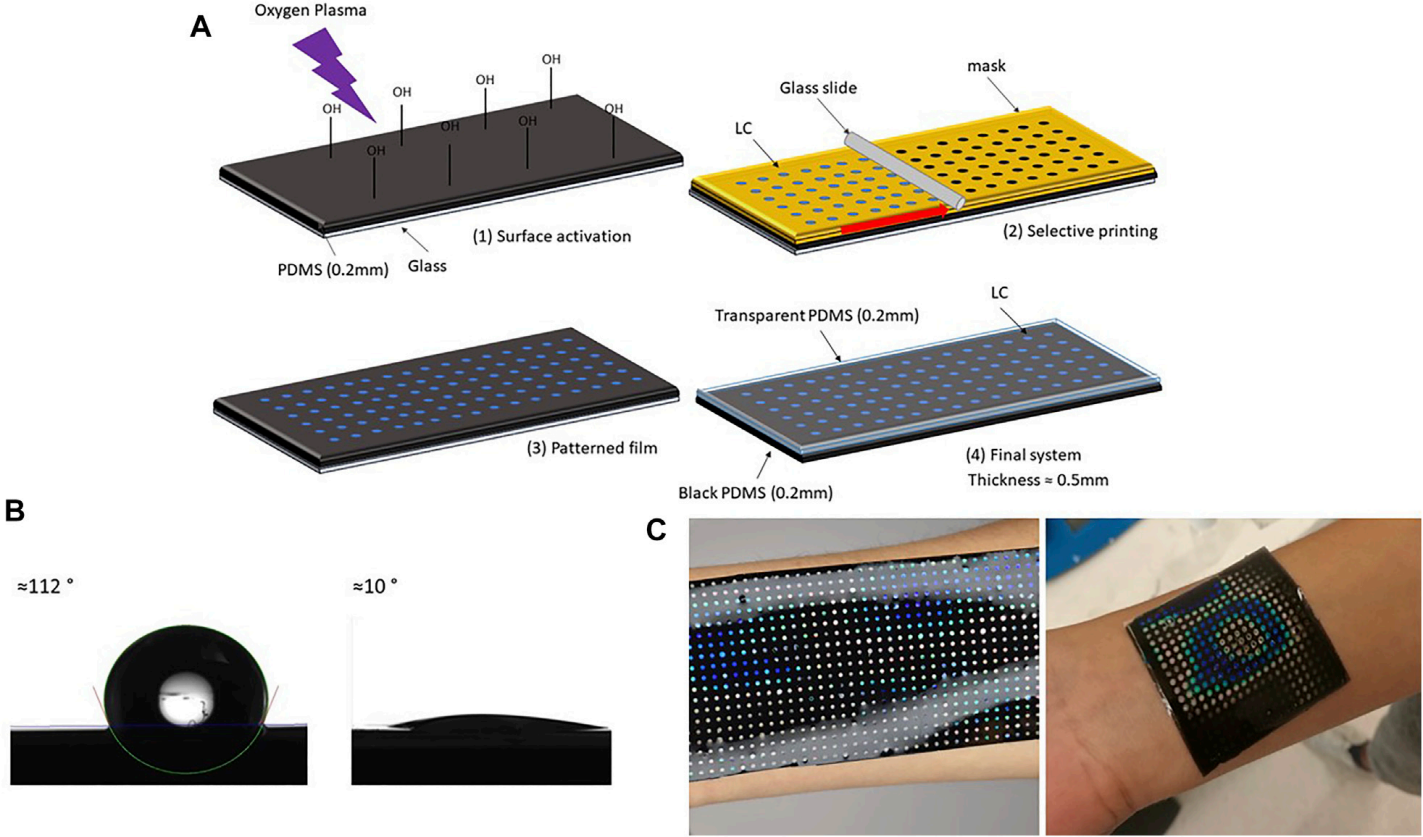
Liquid crystal sensing patch with arrays: production steps. **(A)** Schematic description of LC sensing patch preparation: (1) thin layer (200 *μ*m) of black PDMS (PDMS and CB) was spin-coated on a glass substrate, after curing thin film was transformed into hydrophilic surface using oxygen plasma (2) patterned paper mask (thickness 100 *μ*m) was glued on top of PDMS film, and a drop of LC was poured on top of the mask. A uniform layer was created by blade coating (3) mask was removed when LC cooled down (4) another thin layer of transparent PDMS was spin-coated on top. After curing at the room temperature patch was peeled off from the glass. **(B)** PDMS contact angle before and after oxygen plasma treatment. **(C)** Different forms of LC patches adapting to different body parts.

The liquid crystal sensing element (also referred as sensing patch) consists of three layers: a bottom polydimethylsiloxane elastomer/carbon black (PDMS/CB) layer, a middle LC sensing layer and a top transparent PDMS elastomer (PDMS) layer. The sensing patch was produced following the steps shown in Figure 1A.

The first step in the fabrication process was a production of a thin black bottom elastomer layer. PDMS SYLGARD™ 184 (Dow Inc.) and CB, particle size 4 *μ*m (Nanografi) were used to produce elastomer layers. PDMS components were mixed in a ratio 10:1, with the addition of 1 wt% of carbon black powder to produce the black bottom layer. The mixture was placed under the vacuum for 15 min to remove air bubbles. To make a thin film, PDMS/CB was spin-coated on the glass substrate (50 mm × 50 mm) and cured at 100°C for 30 min. For the glass substrate with 50 mm × 50 mm dimensions, PDMS/CB was spin-coated for 30 s using a speed of 500 rpm. The thickness of the bottom layer was measured using a digital optical microscope (Keyence VHX-6000) and its build in-feature–*Plane measurements between two points*. After the curing sample was peeled from the glass substrate and cut in half and fixed between two acrylic blocks. The fixed sample was placed under the microscope (magnification ×20) in a vertical position to visualise the cross-section. The thickness was then measured ten times across the section, and the average value of approximately 200 *μ*m is calculated. The black bottom layer was chosen for the best visualization of the colours. Thermal conductivity of pure PDMS and PDMS/CB was measured using Hot disk TPS 2500 S. Sample thickness was 5 mm, heating power 20 mW and time 10 s. Thermal conductivity of pure PDMS was 0.19 W/mK, while PDMS/CB had thermal conductivity of 0.18 W/mK. This measurement showed that addition of CB in this small concentration (1 wt%), does not influence the thermal conductivity of PDMS.

The second step was PDMS/CB surface activation with oxygen plasma, to increase the hydrophilicity of PDMS/CB film. Before plasma treatment, PDMS films were cleaned from dust using scotch tape. Films were treated with oxygen plasma using a plasma cleaner (PDC-002 (230 V) Haarrick Plasma) for 15 min, using the maximum power–30 W. The water contact angles before and after the treatment are shown in Figure 1B. An oxygen plasma treatment of 15 min using this procedure increased hydrophilicity of PDMS enough for successful printing. The contact angle before surface treatment was 112°, and after treatment, it became less than 10°.

The third step was an LCs dots array fabrication on the PDMS/CB layer, achieved using the blade coating technique. The plane surface was converted to a grid of dots to obtain the uniform colour response, and this was achieved by blade coating over the patterned paper mask. The mask was designed in CorelDraw, with the final dimensions of 50 mm × 50 mm, containing 324 dots with a diameter of 1.5 mm, and a 1 mm distance between them. A laser cutter (EpilogLaser mini) was then used to cut the paper sheet (thickness 100 *μ*m) into the designed pattern. After removing spin-coated PDMS/CB from the plasma cleaner, the mask was glued on top of the sample, and LCs in liquid form (0.5 g for each patch) were blade coated, using a glass slide, moved by a motor with the speed of 50 rpm. The mask was removed once the LC film cooled down. The goal of the process is to cover as much as possible the original shape to maximize the sensing area, keeping at the same time the high sensibility related to homogeneity. With this configuration, the sensing area was covering approximately 23% of the total one, with 324 homogeneous highly sensitive components.

The final step was to cover LC pattern film with the protective transparent PDMS layer, using the spin coating technique (speed 500 rpm, time 30 s). Digital photos of the patch adapting to the different body surface is shown in Figure 1C.

### 2.4 Experimental Setup

In general, an experimental setup for the calibration of thermotropic LCs should consist of a calibration surface with a temperature sensor, imaging system, heating and cooling system, and illumination source. The experimental setup, shown in Supplementary Figure S1, was used to record the LC patch’s colour changes with the temperature. Peltier elements with an aluminium plate was used as heating and cooling systems. The aluminium plate was used to obtain uniform plate temperatures. Two temperature sensors were within system. A temperature sensor, TS1, was placed below the aluminium plate and was used to control the temperature of the Peltier element, while another temperature sensor, TS2, was placed on top of the aluminium plate. The imaging system includes hardware [colour camera (Jai GO-5000-USB)] and software for colour extraction. A 5500K LED light was used as an illumination system. There are different types of illumination-viewing arrangements. Here, an on-axis arrangement was used. This arrangement was achieved by placing an LED light ring around the colour camera.

### 2.5 Assessment of the Liquid Crystal Sensor Functioning

#### 2.5.1 The Relation Between Temperature, Colour and Hue

In order to use thermotropic LCs for quantitative temperature measurements, the determination of the relation between temperature and colour is a necessary step. Several ways to specify colour include the Red, Green and Blue (RGB), and the Hue-Saturation-Value (HSV) model. Researchers have widely used Hue to quantify colour due to its simplicity and independence with respect to illumination intensity. It is considered that the analysis of HSV data represents the simplest and most straightforward analysis approach. Hue (in degrees °) is what people typically refer to when using the term “colour.” Saturation describes the degree to which a pure colour is diluted with white. It identifies how pure or intense the colour is. The value (brightness) of colour identifies how light or dark the colour is. In the following graphs, Hue will be used to quantify the colour.

This calibration of the patch should be done in an environment as close as possible to the conditions in which it will be used. The calibration was then performed in a laboratory open space so as to simulate normal utilization conditions, e.g., patients at home. Before each test, temperature and humidity in the laboratory were measured.

Before placing the sample on the aluminium plate, an automatic white balance was carried out on the white background to calibrate the colour temperature. Once the sample was placed, TS2 was in direct contact with the bottom of the patch. During the test, the temperature was increased or decreased in the cooling case, with steps of 0.1°C. When the temperature measured with TS2 was stable, photos were taken. Exposure time was set to 10,200 ms and ten pictures for each temperature were taken. RGB values were collected from dots that were in direct contact with the TS2.

#### 2.5.2 Repeatability Test

In general, the sensor’s repeatability is considered to be an important parameter, mainly if the sensor should be used multiple times and be exposed to heating and cooling conditions, like in the case of a sensor that would be used in wound monitoring. To test this property systematically, six different full-range calibrations were performed within 3 days. Each day, the first test was dedicated to heating, and the second test for cooling. Between these calibrations, the patch was stored in ambient conditions. The sample was not moved from its original place during this 3-day period, and hue values were calculated on the same spot.

#### 2.5.3 The Dynamic Test

Response time is another important sensors’ characteristic. In this study, response time was measured using a dynamic test. First, the sample patch was placed on the aluminium plate and then the temperature was set to 34°C. The monitoring started after the system was stabilised, i.e., TS2 temperature and Hue were not changing. During the test, temperature from sensor TS2 was constantly monitored (20 values per second), and images were taken automatically (10 frames per second) using a colour camera. When the monitoring started, the set-point temperature was changed from 34 to 37°C.

#### 2.5.4 Application for Colour/Hue Quantification

**FIGURE 2:**
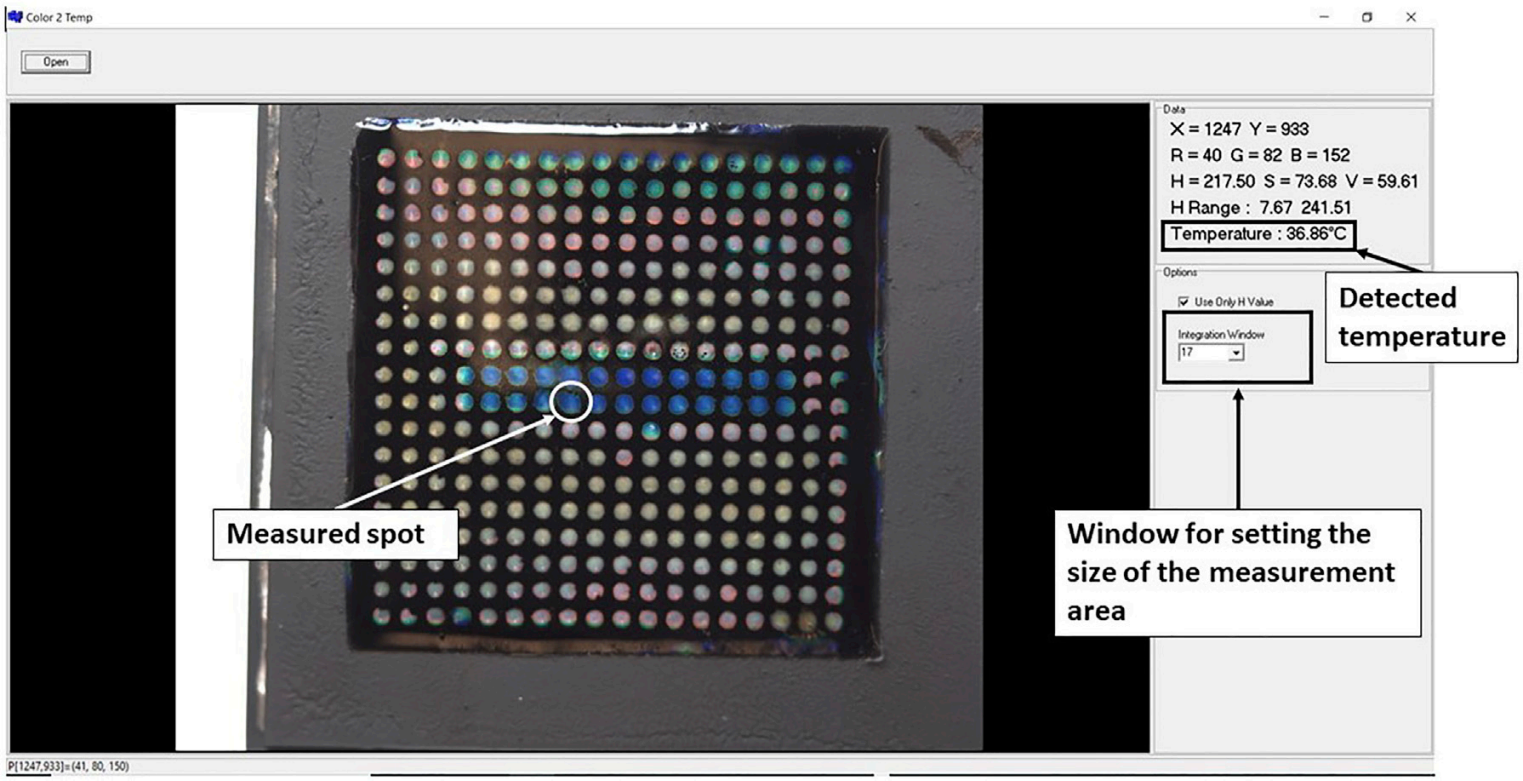
The interface of the application used for quantitative analysis of temperature from the LC patch.

A C++ application was developed to allow quantitative reading of the temperature using the LCs patch. An example of the temperature reading is shown in Figure 2. The size of the selected area can be directly modified in the integrated window. We can choose the number of pixels on each side of the central point, which provides the Hue values for each pixel. From this, the application will then calculate the average hue value converting it to the temperature of the selected area, using the data from the calibration curves (see later in Figure 5).

#### 2.5.5 Comparison With IR Thermography

The goal was to directly compare the temperature distribution of the same surface obtained using the LCs patch with the one recorded using an Infrared Thermal Imaging Camera, FLIR T425 with a 320 × 240-pixel resolution. This test was performed on the same setup used for calibration. In this case, instead of an aluminium plate, we used flat surfaces composed of two materials with different thermal conductivities, aluminium (thermal conductivity 237 W/mK) and an acrylic sheet (0.2 W/mK). Three different surfaces were prepared, where the base was an aluminium plate with different 3D shapes engraved using a CNC machine and opposite acrylic masks. The goal of creating these plates is to get a flat surface that will, during heating, have different temperature distributions on the top. This will mimic the temperature distribution at the wound and surroundings. Digital photos of the surfaces are shown in Supplementary Figure S2. All surfaces were sprayed with conductive black spray to make IR imaging more accurate. Firstly, an LC patch (50 mm × 50 mm) was placed on the heated surface and photos were taken. Following that, the patch was removed, and a corresponding IR images from the same surface were taken. The IR photos were taken at room temperature, and FLIR was left on for at least 10 min for stabilization before imaging. The distance between the FLIR camera and the mould was 0.2 m, and emissivity was set to 0.75. The value for the emissivity was chosen based on the calibration measurements, where two sensors (with accuracy 0.1°C) were placed directly on the surface and the temperature measurements were compared to the IR measurements from the top.

## 3 Results

### 3.1 Choice of LC Formulation

**FIGURE 3:**
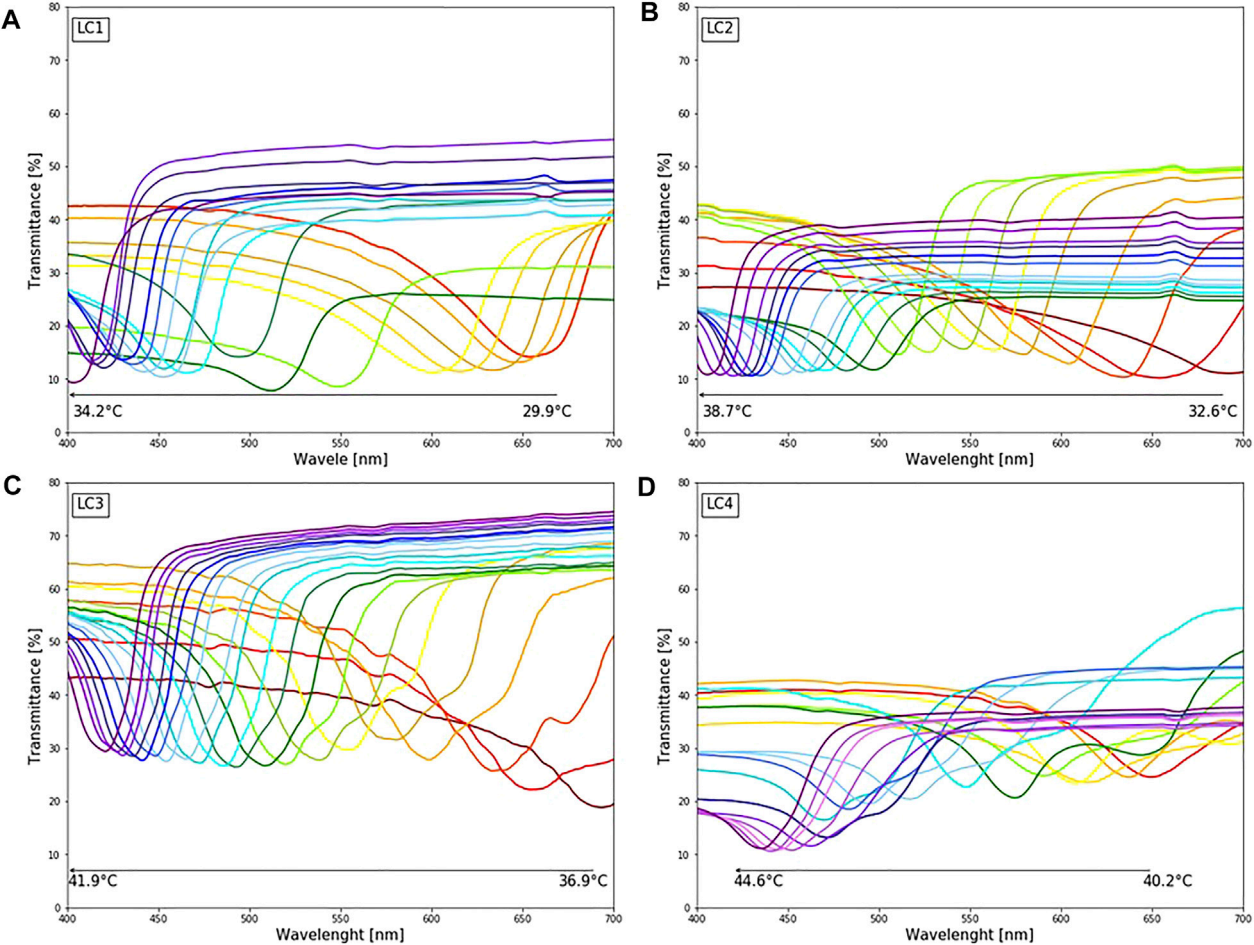
The colorimetric response of different LC upon changing temperature (29.9–44.6°C): **(A)** LC1: reflection peaks in the visible spectrum for temperature range 29.9–35°C **(B)** LC2: reflection peaks in the visible spectrum for temperature range 32.6–38.7°C **(C)** LC3: reflection peaks in the visible spectrum for temperature range 36.9–41.9°C **(D)** LC4: reflection peaks in the visible spectrum for temperature range 40.9–44.7°C.

**FIGURE 4:**
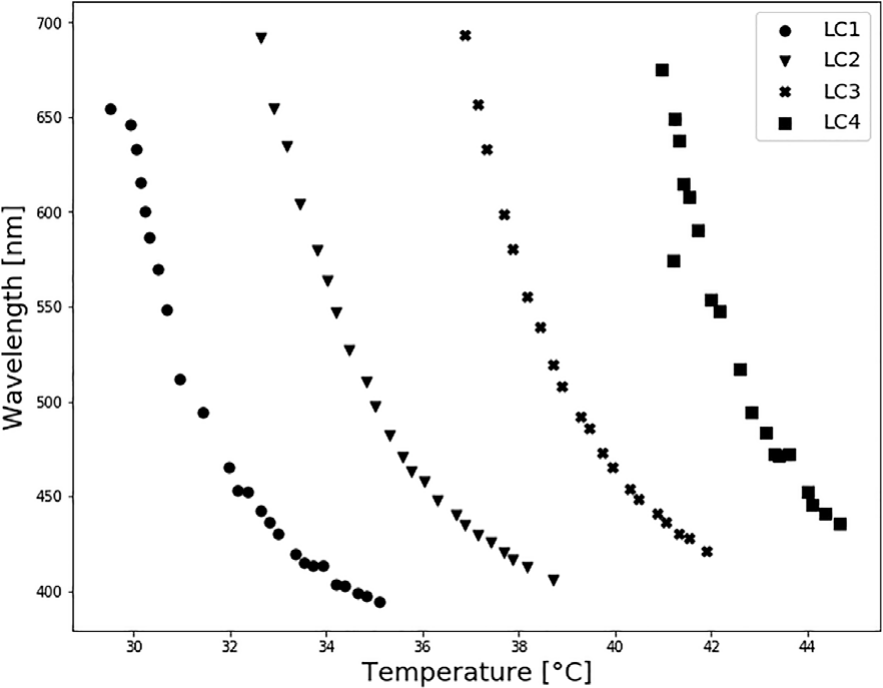
Thermotropic LC spectrophotometry: calibration curves. All calibration curves showed a second-order polynomial trend.

Transmittance spectra of LCs are shown in Figure 3. For each LC formulation, reflection peaks were shifted towards a lower wavelength, i.e., blue region, with temperature increase. Calibration curves were created by taking the reflections’ peak wavelength values for each temperature, presented in Figure 4, to establish which liquid crystals combination was the best for the described application. As expected, different LC formulations showed the optical active range in different temperature ranges, shown in Table 2. On average, the temperature span was 5°C. All calibration curves showed a second-order polynomial trend. For more straightforward representation, calibration curves were divided into two linear regions, higher and lower sensitivity regions. Sensitivity and range for these regions are shown in Table 2. The sensitivity was calculated as the slope of the calibration curve, and it is presented in nm/°C. All formulations showed the same trend, higher sensitivity (78 nm/°C) for lower temperature ranges (red-green colour range) and lower sensitivity (25 nm/°C) for higher temperature ranges (blue colour range). This trend is well-aligned with reports in the literature (Stasiek et al., 2014). Accordingly, for lower temperature ranges, the resolution is 0.013°C. While for higher temperatures the resolution is 0.04°C. Both resolutions are fulfilling requirements for a wound temperature sensor. LC2 system was chosen to produce the sensing patch, since the temperature range (32.5–38.7°C), where pitch corresponds to the wavelength of visible light, is connected to the wound healing temperature range. However, the described procedure can be used to produce patches with any thermotropic LC formulation.

**TABLE 2:** Liquid crystals response range and sensitivity. Measurement done using the spectrophotometer.

Sample Name	Linear response range 1 (°C)	Linear range sensitivity 1 (nm/°C)	*R* ^2^	Linear response range 2 (°C)	Linear range sensitivity 2 (nm/°C)	*R* ^2^
LC1	29.9–32.0	89.7	0.95	32.1–35.1	25.3	0.98
LC2	32.6–35.9	71.2	0.98	36.0–38.7	18.9	0.98
LC3	36.9–39.5	78.6	0.97	36.6–41.9	23.7	0.98
LC4	40.9–43.1	81.6	0.88	43.2–44.7	31.2	0.94

### 3.2 Relation Between Temperature and Hue

**FIGURE 5:**
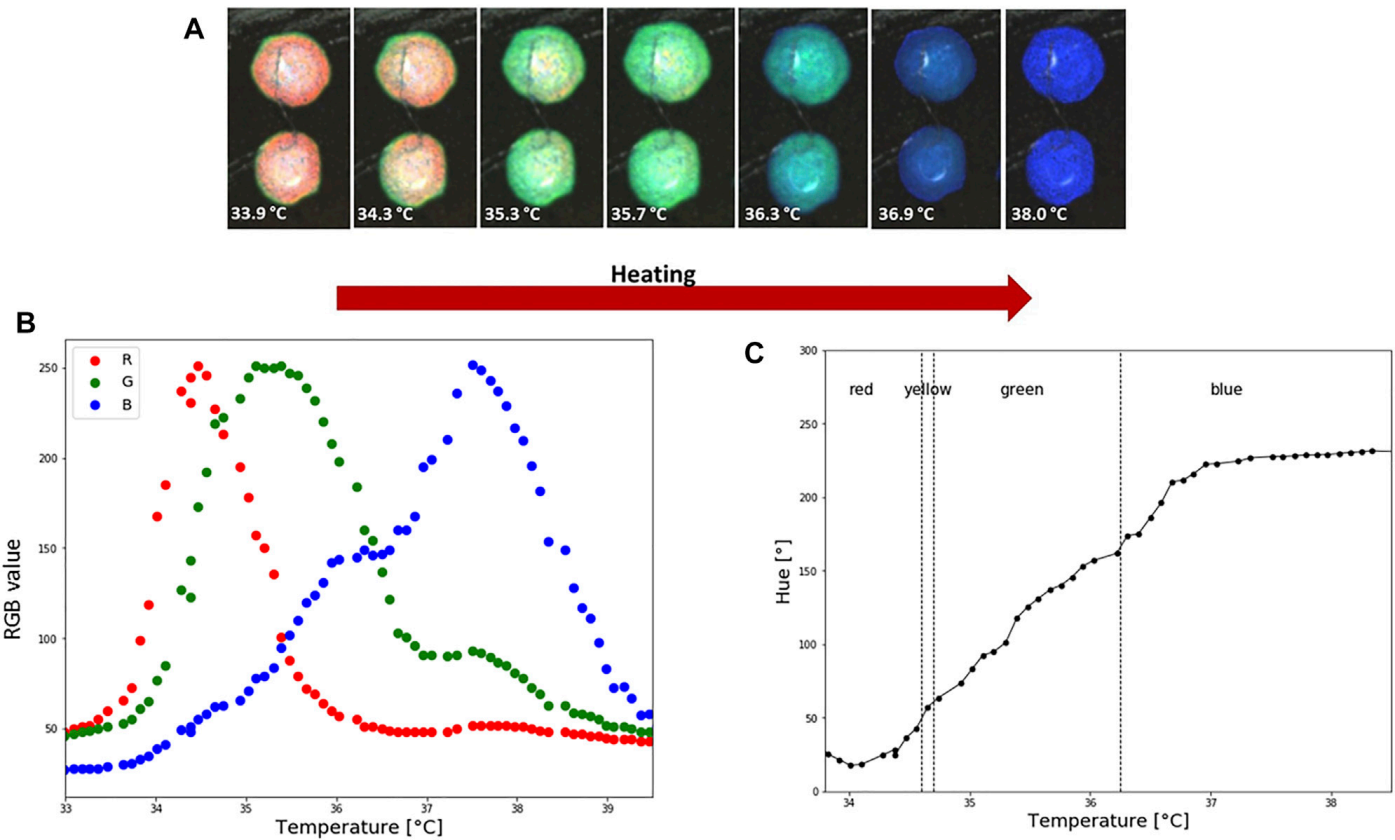
LC2 patch colorimetric response during heating and calibration. **(A)** How the temperature is increasing LC are going through an optical active range, which starts with red and finishes with blue colour. Average RGB and HSV values from two spots were used to create a calibration curve. Each spot is 50 × 50 pixels. **(B)** Temperature and RGB dependence. **(C)** Hue-temperature calibration curve. During the calibration the ambient temperature was 23°C and relative humidity 45%.

Photos used for calibration are shown in Figure 5A. RGB values were taken from two spots, each 50 × 50 pixels, that were in direct contact with sensor TS2. Representative calibration curves, including RGB-temperature dependence and Hue-temperature dependence, are shown in Figures 5B,C. The Hue—temperature relationship is strongly non-linear, as can be seen in Figure 5C. The non-linear trend of Hue-temperature dependency for thermotropic LCs is expected and previously reported in the literature (Sabatino et al., 2000; Anderson and Baughn, 2004). The hue-temperature curve shown in Figure 5C can be well fitted by a 5-order polynomial (*R* ^2^ 0.991), which is used to convert LCs colour images to the temperature distribution. The part where hue increases monotonically with temperature is known as the effective temperature range or hue bandwidth. From Figure 5C it can be seen that the effective temperature range in LC2 is approximately 3.5°C, from 34 to 37.1°C. These types of LCs that have bandwidths within the range of 0.5–4°C are typically referred to as narrowband thermotropic LCs. Although they cover smaller temperature ranges than wideband LCs, the advantage is their higher precision in temperature measurements and are less affected by variations in illumination intensity (Abdullah et al., 2010). Sensitivity in the effective temperature range, corresponding to lower temperatures (34–37.1°C) and red and green hue values, is 73°/°C. However, entering the blue regions (37.1–38.6°C), sensitivity drops to 4.5°/°C.

### 3.3 Repeatability

#### 3.3.1 Temperature Shift in Time

**FIGURE 6:**
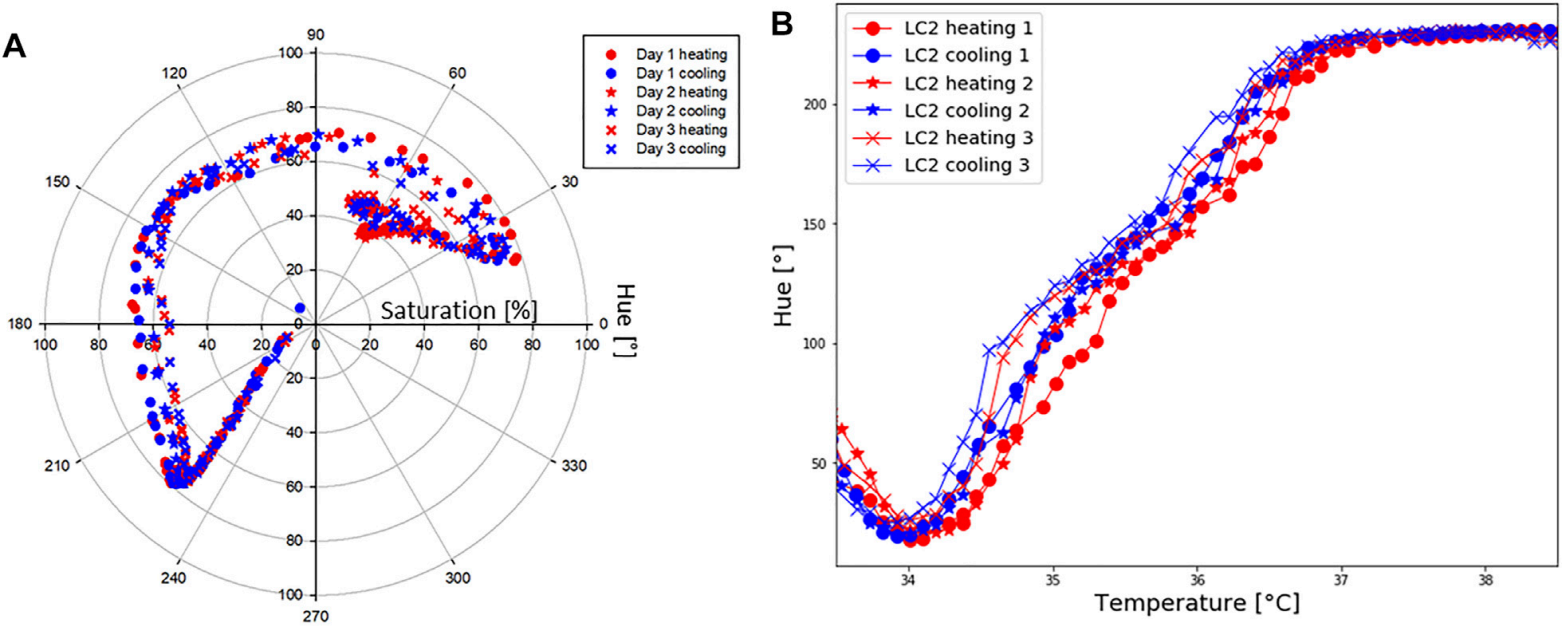
LC2 patch: repeatability. **(A)** Hue [°] and saturation [%] values during six separate tests, done in 3 days. **(B)** Hue [°]-temperature [°C] test performed to study repeatability.

Hue-saturation values are presented in Figure 6A, and Hue-temperature curves on Figure 6B. It is observed from Figure 6A that colours are less saturated as time increases. The temperature shift in the calibration curves at four points (Hue = 50°, 100°, 150° and 200°) is presented in Table 3. Compared to the first calibration curve, the temperature shifts increased with time, and was the highest on the last day during cooling, reaching 0.47°C. The ageing had a maximum effect on the hue-temperature relation for lower hue values. The magnitude of the temperature shift is in agreement with literature, but it shows the opposite trend. Wiberg and Lior (2004) reported a maximum shift of 0.4°C but towards higher temperatures, while shifts towards lower temperatures were observed in our study. In addition, this experiment showed that time is causing the drop in sensitivity, see Table 4.

**TABLE 3:** LC2 patch: Temperature shift (°C) compared to the first heating calibration curve.

Hue (°)	Cooling 1	Heating 2	Cooling 2	Heating 3	Cooling 3
50	0.25	0.09	0.20	0.33	0.47
100	0.24	0.09	0.19	0.29	0.42
150	0.23	0.1	0.18	0.26	0.37
200	0.22	0.1	0.17	0.22	0.32

**TABLE 4:** LC2 patch sensitivity in effective temperature region 34–37.1°C.

Measurement	Sensitivity (°/°C)	*R* ^2^
Heating day 1	72.8	0.99
Cooling day 1	71.8	0.97
Heating day 2	73.1	0.98
Cooling day 2	71.6	0.98
Heating day 3	69.4	0.97
Cooling day 3	67.74	0.97

#### 3.3.2 Hysteresis

**FIGURE 7:**
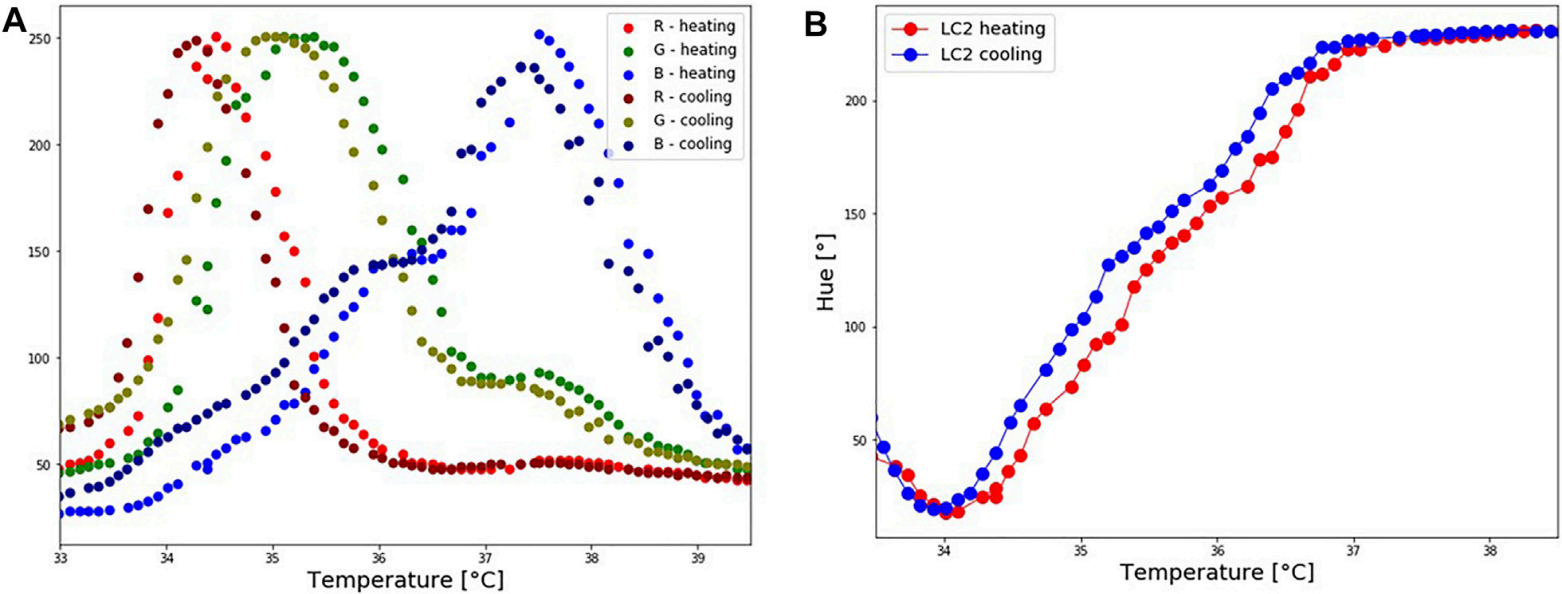
LC2 patch: Hysteresis effect. The patch was first heated until reaching the isotropic phase, and cooled down. **(A)** Shift in RGB values during the cooling process. **(B)** A corresponding shift in Hue values during the cooling process.

During the repeatability test, it was observed that the hue temperature relationship depends upon whether the crystals are undergoing a cooling or heating cycle. This difference is known as the hysteresis effect. The hysteresis effect was studied by heating the patch until 40°C and cooling it down until 32.5°C. Hue was calculated during heating and cooling for steps of 0.1°C. Corresponding RGB-temperature and Hue-temperature curves are presented in Figures 7A,B. In the cooling cycle, a shift in R, G and B peaks is observed, resulting in a shift to a higher hue for the same temperature. The hysteresis effect was the highest in the temperature range around 35°C, whereas for the same hue value the difference in the temperature was approximately 0.26°C. Several other studies (Dixon and Scala, 1970; Zink and Belyakov, 1997; Sabatino et al., 2000; Anderson and Baughn, 2004; Kakade et al., 2009) reported a hysteresis effect in thermotropic LCs. These studies observed that the hysteresis effect appears if LCs are heated above their clearing point.

### 3.4 Dynamic Test–Response Time

**FIGURE 8:**
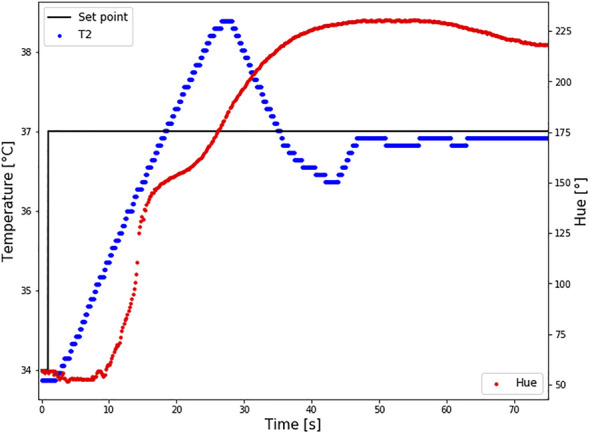
Response time of LC2 patch. The set point temperature was changed from 34 to 37°C (black line), and the temperature sensor TS2 measured the temperature at the bottom of the patch (T2-blue curve). At the same time changes in Hue were calculated (red curve). Intimidate reaction in hue changes how the temperature is changing was reported.

Changes in temperature and hue are presented in Figure 8. The first change in Hue was detected 0.25 s after the first changes in temperature, which indicates an almost immediate response of hue on temperature colour. This response cannot be clearly seen in the graph because the first change occurred in the red region, before the effective temperature region. The response time is the same in both the heating and cooling cycles.

### 3.5 Comparison Between LCs Wearable Patch and IR Imaging

**FIGURE 9:**
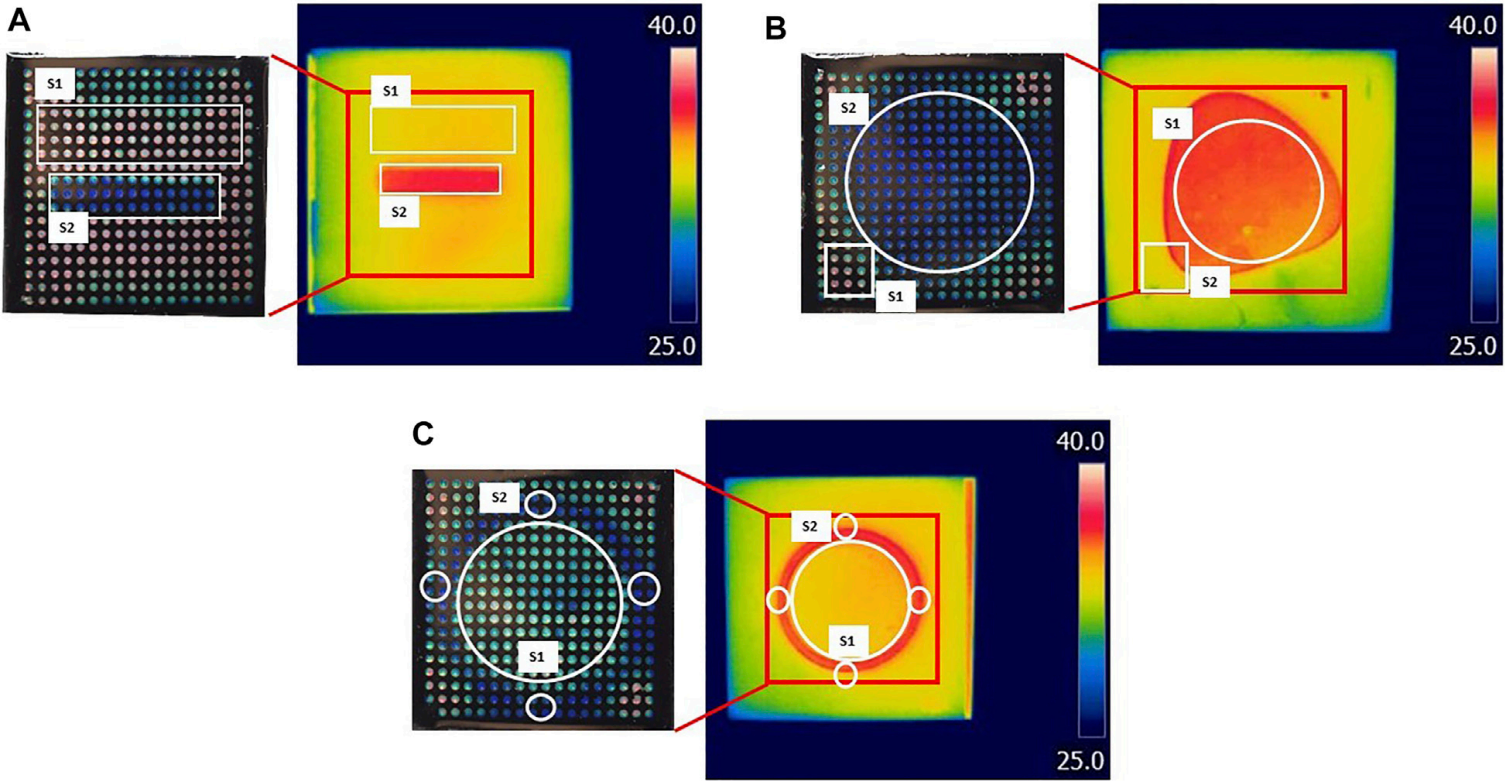
Comparison between LCs patch and IR imaging. Three surfaces **(A–C)** with different temperature distributions were tested. On the left are digital photos of LCs patch response when in contact with the heated surface; on the right are IR images of the same surface. The temperature was calculated from the surfaces marked with white on both digital and IR images. The red square on IR images represents the surface covered by the LC patch.

Results are shown in Figure 9; Table 5. Although a relatively accurate temperature distribution of the surface using the LC paths was achieved, the pattern recognition and sensing quality were higher using the IR camera. ΔT was calculated for IR and digital images for each shape (see Figure 9), as the difference between the average temperature of the hotter and colder surface. The difference in ΔT, calculated using IR and LC images varied for different shapes. The highest difference was observed for shape 1, where the difference between the two measurements was 0.7°C. In contrast, there was no difference between IR and LC techniques for shape 3. This phenomenon can be contributed to the quality of the contact between the surface and the bottom of the patch. Although the results are encouraging, some improvements are in order, which will be discussed in the following section.

**TABLE 5:** Comparison between temperature measurements using LCs patches and application against the ones measured using IR imaging.

Measured area			Tmax [°C]	Tmin [°C]	Taverage [°C]	ΔT [°C]
Shape1	IR	S1	34.8	32.8	33.9	2.6
		S2	37	35.8	36.5	
	LC	S1	36.8	33.2	35.4	1.9
		S2	38.4	36.5	37.3	
Shape2	IR	S1	34.6	32.1	32.8	3.1
		S2	37.5	31.6	35.9	
	LC	S1	36.5	33.1	34.9	2.5
		S2	38.8	36.3	37.5	
Shape3	IR	S1	35.8	33.6	34.4	1.8
		S2	36.4	35.9	36.2	
	LC	S1	36.5	34.0	36.1	1.8
		S2	38.3	36.6	37.9	

## 4 Discussion

As described in the introduction, IR thermography is commonly used to measure temperature distributions in wound studies. IR thermal cameras are sensitive to environmental conditions. Before imaging, IR cameras require input values including room temperature, humidity, distance from the object and its emissivity. It is not always possible to correctly determine these parameters, although they are directly influencing the reading output. The emissivity of human skin is considered to be between 0.97–0.99 (Boylan et al., 1992; Keenan et al., 2017), while different studies have found that the emissivity of wounds can be greater by 0.01–0.03. This could result in an underestimation of the ΔT value by 0.1–0.2°C (Boylan et al., 1992), and could prove significant in clinical evaluations of some studies. Depending on the location and size of the wound, the temperature of the healthy surrounding skin can vary for several degrees (Carriere et al., 2020). Hence directly influencing the ΔT value and the reading outcome. Moreover, the wound bandage should be removed for each temperature measurement using IR thermal cameras. This is a disadvantage because it prevents continuous temperature monitoring.

In general, this study showed that LCs have the potential to fabricate advanced temperature distribution sensors. This is not common to other sensing elements, such as resistance or impedance-based ones, that could measure only point-wise values. Another important advantage of this configuration is its simplicity in reading the output. When compared with infrared imaging, LCs are capable of reproducing the main features of the temperature fields surrounding a wound mimicking shape, even geometrically complex. However, the recognition of the patterns was somewhat less sharp for the LCs with respect to the IR camera. This might be caused by the contact between the patch and the to-be-measured surface.

The thermotropic LCs used in this research can detect even small temperature variations (high sensitivity) and have a good repeatability as well as a fast response time (less than 0.5 s). However, LCs used here have a narrow bandwidth, resulting in high sensitivity but a small effective temperature range. The temperature range can be extended by changing the type of thermotropic LCs or by changing the component ratio. According to (Ochoa et al., 2014), the preferred requirements for temperature wound monitoring sensors depend on the wound’s type. However, during the wound healing process, the dynamic temperature range is 25–41°C. The other formulations (see Figure 4), showed that it is possible to cover from 29–44°C. In this study only one of them was used in order to focus on the principle and show the feasibility of such an approach, but the same study can be performed on the other liquid crystals.

The ageing of LCs reduces their shelf life. Proper preparation and storage are necessary to minimize their ageing. It was suggested that pure thermotropic LCs should not be exposed to fats, greases, organic solvents and dust. They can also be susceptible to UV and IR radiation, and exposure to these sources reduces the shelf life (Abdullah et al., 2010). Another observation is that the hue–temperature dependence is non-linear. The decrease in sensitivity is particularly notable when entering in the blue region, where small changes in temperature do not cause significant changes in hue. The cause of hysteresis in cholesteric LC is complex. Previously it was suggested that hysteresis is strongly associated with chemical composition (Dixon and Scala, 1970) and that hysteresis depends on the sample thickness (Zink and Belyakov, 1997). In the case of a narrowband LC, the thicker sample showed no hysteresis, and the thinner sample showed up to about a 0.5°C bias toward lower temperatures when cooled (Zink and Belyakov, 1997). Moreover, the magnitude of the shift increases with increasing maximum temperature before cooling. For this particular application, hysteresis should be avoided since it can give false information about temperature at the wound during cooling. Hysteresis can be reduced significantly if the operating temperature is regulated. Most importantly, the operating and storage temperature should be kept below the clearing point temperature (Zink and Belyakov, 1997). However, this does not necessarily represent a problem if the calibration is done with small steps (0.1°C) and a computerised way of reading colour is included, such as the application used in this work.

Note that the technological readiness of LCs, although promising, does not directly translate into the possibility to include them in clinical trials. Several issues should be considered in that respect and they will be discussed below.

Wound contamination: the current status of the proposed patch requires that it should be in direct contact with the wound. In clinical practice, medical personnel are obliged to use wound dressings to avoid contamination, protect wounds, and promote wound healing. Nowadays, an ideal wound should fulfil the following characteristics: 1) creating a moist and warm environment around the wound, 2) allowing gas exchange, 3) protecting the wound from bacterial infections, 4) creating a mechanical protection, 5) controlling the exudate level, 6) being biocompatible, non-toxic, non-allergenic, 7) being easily removable, i.e., the material should be non-adhering to avoid removing of a newly formed tissue, 8) being able to stimulate healing, and 9) being costly acceptable (Sood et al., 2014). Applying the proposed LC system on top of the wound dressing could influence some of the aforementioned dressing’s characteristics, such as gas exchange, exudate and moist control properties. Moreover, the LCs sensing characteristics could be affected by the micro-environment of the wound and surroundings. Another option would be to use the LC-based sensor just during the bandage removal. In this case, continuous monitoring is not possible.

Size of the wounds: in literature it is possible to find an outstanding number of wound sensors, either based on LCs or not. Everyone who participated to a real clinical study on wound imaging knows that serious wounds, and especially burns, could cover large portions of the body, sometimes even the whole body. It is evident that the development of a contact sensor that would monitor such a wound is currently not possible.

Shape and 3D nature of the wounded tissues distributions: although maybe technically possible, it is very difficult to imagine a contact sensor when the shape and distribution of the wounds is not flat or even worse, changing “volumetrically” during the healing time. It would be possible to think of an intermediate layer hosting the sensing element. If this solution could potentially solve the flatness problem, it will also challenge the capability of this layer to transmit reliably biophysical signals from the wound to the sensing element.

Although it is beyond the scope of this paper to provide a complete overview of the clinical requirements for wound diagnosis and follow-up, it is possible to draw preliminary conclusions. LCs could represent a complementary element to support the medical decision and follow-up in the case of wounds, especially burns. However, the application of such kinds of sensors in clinical practice is far from being straightforward, and the plethora of sensors’ concepts presented in the literature are still at a low-TRL stage.

## Data Availability

The original contributions presented in the study are included in the article/Supplementary Material, further inquiries can be directed to the corresponding author.

## Nomenclature

LC: liquid crystal
IR: Infrared
CB: carbon black
TS: temperature sensor
