# Supplementary material for: Thermotropic Liquid Crystals for Temperature Mapping
Source: Front Bioeng Biotechnol. 2022 May 12;10:806362. doi: 10.3389/fbioe.2022.806362 (PMC9133408; doi:10.3389/fbioe.2022.806362)
Supplement: Supplementary file 1 [file DataSheet1.PDF]

Supplementary Material

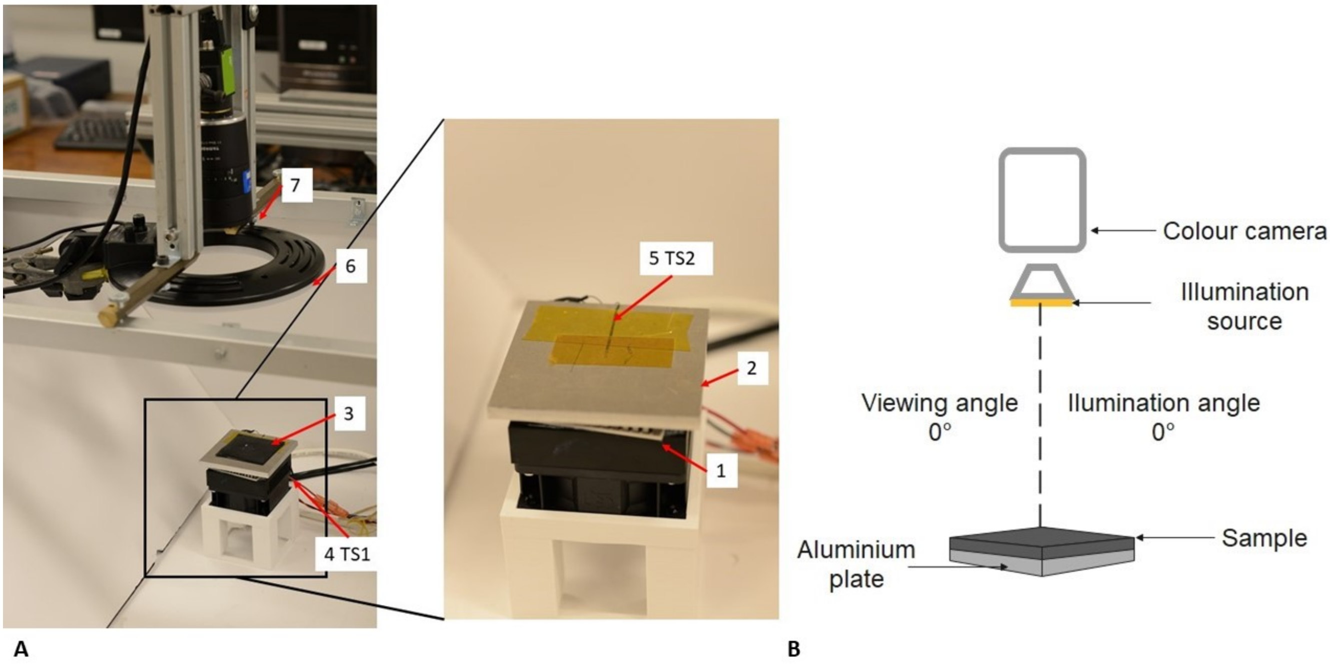

Figure S1. Experimental setup for the patch characterization: **(A)** Digital image: 1 - Peltier element, 2 - aluminium plate, 3 - sample, 4 - temperature sensor TS1, 5 - temperature sensor TS2, 6 - light source, 7 - colour camera, **(B)** Schematic representation.

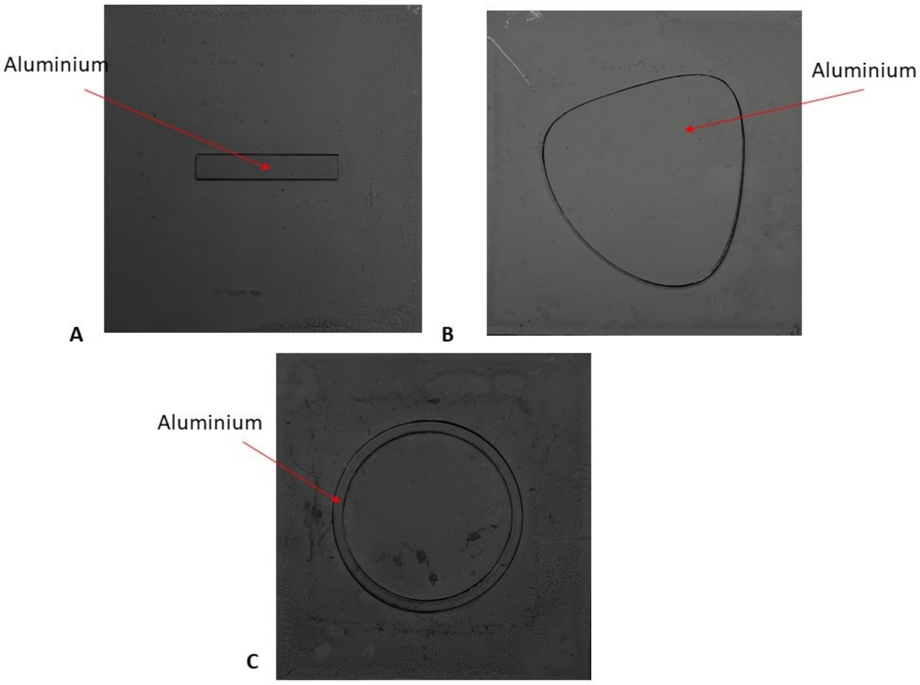

Figure S2. Digital images of three different surfaces created using two materials with different thermal conductivity; aluminium and Poly (methyl methacrylate).
